# Supplementary material for: Assessing the impact of environmental laws and technological advancements on carbon dioxide mitigation in China’s mining industry
Source: PLoS One. 2025 Jun 4;20(6):e0318030. doi: 10.1371/journal.pone.0318030 (PMC12136436; doi:10.1371/journal.pone.0318030)
Supplement: Attachment — Submitted filename: Dr Gilani Review.docx [file pone.0318030.s001.docx]

**Assessing the Impact of Environmental Laws and Technological Advancements on Carbon Dioxide Mitigation in China's Mining Industry**

1. Clarity of Research Objectives: The article sets an important objective to evaluate the impact of environmental regulations and technological advancements on carbon mitigation in China's mining industry. However, the research objectives could be made more specific. It would be beneficial to clearly outline the key questions the study aims to answer and how these align with existing gaps in the literature.

2. Literature Review: While the article references several important works on environmental regulations and carbon mitigation, it would benefit from a more comprehensive review of recent developments in both international and Chinese mining sectors. For instance, it would be helpful to include a discussion on how international treaties, like the Paris Agreement, influence China's domestic policies and technological innovations in the mining sector.

3. Methodology: The article does not provide sufficient details regarding the methodology used to assess the impact of environmental laws and technology. A clear explanation of the data sources, selection criteria for technological advancements, and any empirical or qualitative methods employed would strengthen the overall rigor of the study.

4. Integration of Environmental and Technological Aspects: While the article discusses both environmental laws and technological advancements, the connection between the two remains somewhat disjointed. The study could be improved by explicitly linking how specific technological innovations in the mining industry are directly influenced by environmental policies and how these, in turn, contribute to carbon dioxide mitigation.

5. Case Studies and Examples: To enhance the article’s practical relevance, it would be useful to include more case studies or real-world examples from China's mining industry. For instance, specific technological interventions (e.g., carbon capture and storage technologies, or renewable energy integration in mining operations) and their measurable impacts on carbon mitigation should be discussed in greater depth.

6. Quantitative Analysis: The article could benefit from a more robust quantitative analysis. Incorporating statistics on carbon emissions reduction rates, technological adoption rates, and the effectiveness of environmental laws could provide a clearer picture of the mining industry's progress toward carbon mitigation.

7. Discussion and Policy Recommendations: While the article identifies important trends in China’s efforts to mitigate carbon emissions, the discussion would be stronger with a more in-depth analysis of the challenges and opportunities ahead. What policy recommendations can be made based on the findings? Are there any barriers to technological adoption in the mining industry that need addressing? These considerations would add depth to the conclusion.

8. Conclusion: The conclusion effectively summarizes the study, but it could be improved by more directly addressing the research question. It would also be helpful to outline potential future areas of research, particularly in the context of evolving technologies and tightening environmental regulations in China.

Overall, the article is a valuable contribution to understanding carbon mitigation in China’s mining industry but would benefit from more specific data, case studies, and a stronger linkage between the environmental and technological aspects discussed.
