# Supplementary material for: Assessing the impact of environmental laws and technological advancements on carbon dioxide mitigation in China’s mining industry
Source: PLoS One. 2025 Jun 4;20(6):e0318030. doi: 10.1371/journal.pone.0318030 (PMC12136436; doi:10.1371/journal.pone.0318030)
Supplement: Attachment — Submitted filename: Review.docx [file pone.0318030.s003.docx]

Dear Editor

Dear Editor,

I am writing to confirm that the article titled **"Assessing the Impact of Environmental Laws and Technological Advancements on Carbon Dioxide Mitigation in China's Mining Industry"** has been thoroughly reviewed and is deemed suitable for publication.

This article offers valuable insights into the intersection of environmental policies and technological innovations within China's mining sector, contributing significantly to the academic and practical understanding of carbon dioxide mitigation strategies.

Please proceed with the necessary steps for its publication. Should you require any additional information or support, feel free to reach out.

If there are any further requirements from my side, feel free to reach out.

Best regards,
Dr Syed Raza Shah Gilani
